# Polyurethane-Based Composites for Flexible Sensors: A Review

**DOI:** 10.3390/polym18101254

**Published:** 2026-05-21

**Authors:** Yang Yang, Chao Sun, Xing Zheng, Xinyu Li

**Affiliations:** 1 College of Engineering, Materials and Chemical Engineering, Yanbian University, Yanji 133002, China; 2025050044@ybu.edu.cn; 2 Department of Physics, Jilin University, Changchun 130012, China; sunc344@jlu.edu.cn; 3 Normal College, Yanbian University, Yanji 133002, China; 4 Department of Chemistry, College of Science, Yanbian University, Yanji 133002, China; 5 Department of Polymer Materials & Engineering, College of Engineering, Yanbian University, Yanji 133002, China

**Keywords:** polyurethane, polymer composites, flexible sensors, synthesis, fillers

## Abstract

The rapid advancement of flexible electronics technology has endowed flexible sensors with significant application potential in fields such as wearable sensors, bionic skin, and human–machine interaction, owing to their excellent conformability, stretchability, and comfort. However, as application scenarios continue to expand and deepen, higher requirements are imposed on sensor performance in terms of sensitivity, stability, biocompatibility, environmental friendliness, and multifunctional integration. Polyurethane composites, leveraging their intrinsic characteristics, including tunable molecular structure, superior flexibility, and good biocompatibility, can effectively impart properties such as electrical conductivity, self-healing capability, and high sensitivity through compositing with various functional materials, thereby precisely aligning with the diverse demands of next-generation flexible sensors. This article systematically reviews the synthesis strategies of polyurethane composites; provides a detailed analysis of the roles of fillers—including carbon-based materials, polymers, and metal nanoparticles/nanowires—in enhancing the mechanical, electrical, and functional properties of the composites; and further summarizes the research progress of polyurethane composite-based flexible sensors in cutting-edge areas such as eco-friendly sensing, human motion monitoring, health monitoring, and bionic electronic skin. Future development trends are also discussed, aiming to provide insights for the design and development of high-performance flexible sensors.

## 1. Introduction

Polyurethane (PU) is a class of polymer materials synthesized via the addition polymerization of isocyanates and polyols [1]. The hard and soft segments in its structure can be flexibly tuned, endowing the material with excellent flexibility, abrasion resistance, biocompatibility, and processability [2,3]. As a versatile polymer matrix, polyurethane not only exhibits good mechanical properties and chemical stability [4] but also allows for performance customization through molecular design [5], leading to its widespread applications in coatings, foams, elastomers, and other fields [6]. In recent years, with advancements in materials science, polyurethane has been further endowed with new functionalities such as electrical conductivity, sensing, and self-healing through composite modification, making it a key research focus in the field of flexible sensors [7].

With the rapid development of technologies such as eco-friendly sensors, human motion monitoring sensors, health monitoring, and bionic electronic skin, the demand for flexible sensors is growing. Compared with traditional rigid sensors, flexible sensors need to adapt to more complex curved surfaces, dynamic deformations, and human comfort requirements. Therefore, developing flexible sensors with high sensitivity, good flexibility, reliable stability, and biocompatibility has become a research hotspot [8,9]. Flexible sensors must maintain stable electrical responses under various deformations, including stretching, bending, and compression, while also incorporating characteristics such as lightweight construction, breathability, and even degradability, to meet the dual demands of device performance and wearing experience in practical application scenarios [10,11].

This paper systematically reviews the synthesis methods of polyurethane composites, including techniques such as in situ polymerization, solution blending, electrospinning, and hot pressing. From the perspective of composite materials, it elaborates on the roles and performance characteristics of carbon-based materials (graphene, carbon nanotubes, carbon fibers), polymers (rubber, polyvinyl alcohol, cellulose), and metal nanoparticles in combination with PU. Finally, it summarizes the current application status of polyurethane-based sensors in fields such as eco-friendly sensing, human motion monitoring, health monitoring, and bionic electronic skin and provides an outlook on future development trends, aiming to offer references for research and development in the application of polyurethane composites in flexible sensors.

## 2. Synthesis of Polyurethane Composites

The preparation methods of polyurethane composites are critical to their properties and applications, with different techniques imparting distinct physicochemical and mechanical characteristics. Optimizing the preparation process enables the development of polyurethane composites with enhanced performance to meet specific application requirements [12,13]. The advantages and limitations of various synthesis methods are summarized in Table 1.

### 2.1. In Situ Polymerization

In situ polymerization is a fabrication technique in which monomers, initiators, or catalysts are introduced into a matrix material during composite preparation. By controlling reaction conditions such as temperature, pressure, and time, the monomers undergo direct polymerization within the matrix, forming a polymer and creating a composite structure integrated with the matrix material [12]. The core advantage of this method lies in its ability to achieve strong interfacial bonding between the polymer and the matrix, thereby significantly enhancing the overall performance of the composite [13].

**Figure 1:**
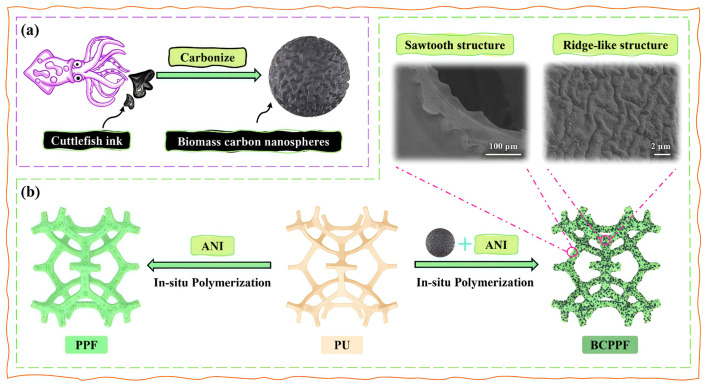
Schematic diagram of the fabrication process of BCNP and PU conductive foam. (**a**) Preparation of the biomass carbon nanospheres; (**b**) Preparation of the BCPPF. Reprinted with permission from Ref. [15]. Copyright 2025 Elsevier.

Li et al. [14] fabricated polyurethane composites with a three-dimensional, dynamic, cross-linked network structure by employing boronic ester bonds as the primary network, hydrogen bonds as the secondary network, and B–N coordination bonds as the tertiary network, using carbon nanotubes and graphene nanoplatelets as conductive fillers. The resulting material exhibited ultrahigh fracture energy, high tensile strength, high elongation at break, high electrical conductivity, high sensitivity, and excellent recyclability. Yang et al. [15] constructed a continuous and stable conductive network by encapsulating biomass carbon nanospheres (BCNPs) and polyaniline (PANI) onto a polyurethane foam framework through in situ polymerization of aniline (ANI). Fluctuations in the solution induced a wavy morphology in the BCNPs/PANI conductive layer, forming a serrated and ridged network structure within the polyurethane foam composite called BCPPF, as illustrated in Figure 1. The developed BCPPF demonstrated high sensitivity, rapid response and recovery times, excellent flexibility and mechanical compressibility, and outstanding stability. Zahra et al. [16] performed in situ polymerization of polyethylene glycol and toluene-2,4-diisocyanate at 40–60 °C, incorporating organically modified montmorillonite (OMMT) into the PU matrix via the cationic salt of 2,2-bis[4-(4-aminophenoxy)phenyl]propane. This approach enhanced the mechanical properties and thermal stability of the polyurethane nanocomposites, yielding high-strength PU composites.

In situ synthesis offers notable advantages in the preparation of polyurethane composites, yet it also presents certain challenges. Its benefits include straightforward synthesis, readily available raw materials, and the ability to form three-dimensional dynamic cross-linked networks through the introduction of boronic ester bonds, hydrogen bonds, and B–N coordination bonds. These structural features endow the resulting composites with high tensile strength, excellent electrical conductivity, and ease of recyclability. Specifically, the materials can achieve ultrahigh fracture energy, high conductivity, and high sensitivity, while maintaining effective monitoring capability even after self-healing and recycling. However, this method also has limitations. For instance, the compatibility between polyurethane and conductive fillers is often poor, and composites incorporating a single-filler system tend to exhibit relatively low electrical conductivity and tensile strength. Moreover, as the proportion of conductive fillers increases, carbon nanotubes are prone to agglomeration, forming linear aggregates that can induce crack propagation under tensile deformation, thereby compromising the mechanical properties of the material.

### 2.2. Solution Blending

Solution blending is an effective strategy for preparing polymer composites. Its core principle involves dissolving the polymer matrix and the desired fillers (such as nanoparticles) together in a suitable solvent to form a homogeneous solution, followed by solvent removal via evaporation or other methods, thereby obtaining a composite with uniformly dispersed components at the microscopic scale [17].

**Figure 2:**
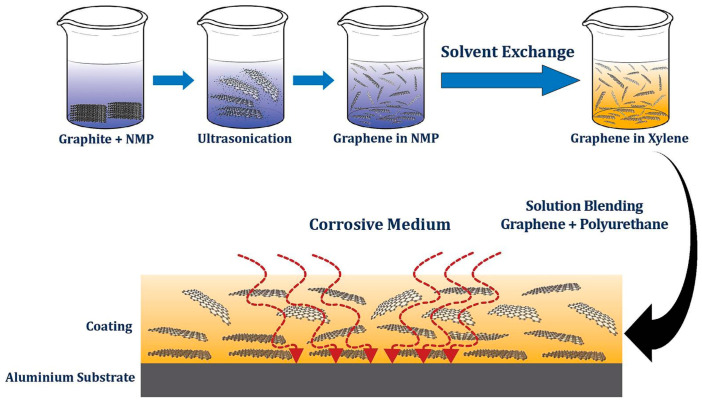
Schematic diagram of the fabrication process of (PU/G) composites. Reprinted with permission from Ref. [19]. Copyright 2024 Elsevier.

Kim et al. [18] dissolved polyurethane and PEDOT: PSS (poly(3,4-ethylenedioxythiophene): polystyrene sulfonate) separately in dimethylformamide (DMF), prepared films via solution blending and drop-casting, and subsequently subjected them to heat treatment. The resulting PU composites exhibited an elongation at break of up to 350%, high self-healing capability, excellent recyclability, moderate electrical conductivity (10 S cm^−1^), and a toughness of 24.6 MJ m^−3^. Pham et al. [19] mixed polyether polyol, crown ether HX, xylene, and graphene flakes using a mechanical stirrer, followed by ultrasonic treatment to obtain polyurethane/graphene (PU/G) composites, as illustrated in Figure 2. The resulting PU/G composites demonstrated long-term anti-corrosion performance, along with significantly enhanced impact strength (124 kg·cm) and adhesion strength (approximately 2.3 MPa). Ali et al. [20] employed solution blending to effectively disperse graphene nanoplatelets into the polymer matrix, promoting uniform interactions between polymer chains and graphene sheets. This facilitated graphene integration and improved interfacial bonding, ultimately yielding polyurethane composites with stable structure and tunable properties. The resulting composites exhibited thermal stability, dielectric performance, and electromagnetic absorption capabilities.

Solution blending exhibits notable advantages in the synthesis of polyurethane composites, enabling the fabrication of materials with electrical conductivity, excellent stretchability, high toughness, and superior mechanical and electromagnetic absorption properties. Furthermore, this method imparts excellent recyclability to the materials and, through precise control of process parameters, allows for the preparation of bubble-free homogeneous films. However, this approach also presents certain limitations. For instance, the mechanical self-healing capability is temperature-dependent; to ensure miscibility between PEDOT: PSS and the PU solution, solvents such as DMF may be required, potentially introducing solvent residue issues. Additionally, the concentrations of additives and polymers must be strictly controlled—exceeding 2 wt% of polyethylene glycol 400 (PEG 400) leads to oily residue formation on the film surface, while excessively low PU concentration (8 wt%) results in poor stretchability, and overly high concentrations (25 wt% and 30 wt%) reduce electrical conductivity without significantly improving mechanical properties.

### 2.3. Electrospinning

Electrospinning is a technique for fabricating micro- to nano-scale fibrous materials from polymer solutions or melts under a high-voltage electrostatic field [21]. This method has garnered widespread attention due to its ability to produce ultrafine continuous fibers with high porosity, large specific surface area, and controllable morphology [22,23]. As an effective approach for preparing nanofibers, electrospinning can be employed to fabricate polyurethane composites with specialized structures and properties.

**Figure 3:**
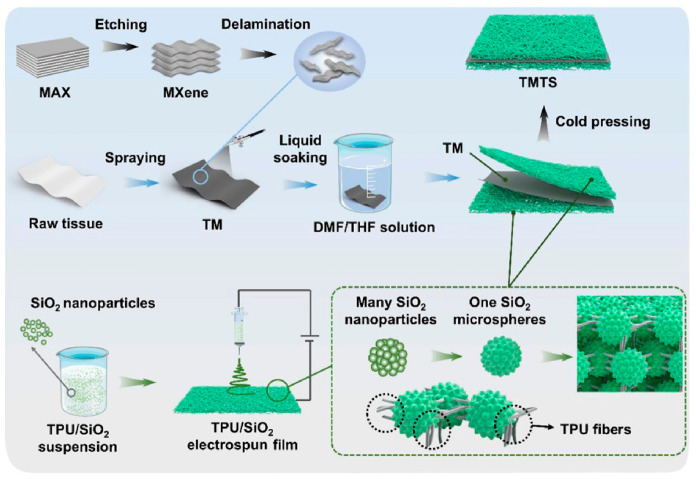
Schematic diagram of the fabrication process of core-shell nanofiber/polyurethane composites. Reprinted with permission from Ref. [25]. Copyright 2025 ACS Publications.

Meng et al. [24] fabricated core-shell nanofiber/polyurethane composites with an interwoven nanofiber network via electrospinning. This unique microstructure provides pathways for energy transmission and charge transfer and enhances multiple scattering of microwaves within the material, endowing the product with excellent electromagnetic wave absorption performance. Huang et al. [25] prepared thermoplastic polyurethane (TPU)/SiO_2_ micro-nanosphere films mimicking the hierarchical surface structure of taro leaves through electrospinning. Subsequently, through systematic optimization of multi-layer assembly processes and techniques, they synthesized superhydrophobic tissue/MXene/TPU/SiO_2_ paper-based composites, as shown in Figure 3. This distinctive hierarchical structure imparted superhydrophobicity (water contact angle of up to 157°, sliding angle as low as 6.5°), stability, self-cleaning capability, high breathability (up to 56.5 g·m^−2^·h^−1^), and antibacterial properties to the composite. Tanalue et al. [26] employed electrospinning to stretch polymer solutions under a high-voltage electrostatic field, skillfully incorporating MXene and ZIF-8 nanoparticles into a polyacrylonitrile/bio-based polyurethane (PAN/PU) matrix. This approach successfully produced composite separators featuring high electrolyte uptake, high porosity, high ionic conductivity, excellent electrochemical stability, thermal stability, and flame retardancy.

Electrospinning offers significant advantages in the preparation of polyurethane composites, primarily reflected in its ability to precisely control the microscopic morphology of materials, thereby fabricating fibrous composites with tunable roughness and high porosity. This endows the materials with exceptional superhydrophobicity, self-cleaning ability, high breathability, antibacterial properties, and outstanding electromagnetic wave absorption performance. Additionally, this method enhances the flexibility and mechanical strength of the composites while offering environmentally friendly processability. However, electrospinning also presents certain limitations. For instance, the sensitivity of the composites may be compromised due to the influence of certain components; the introduction of specific additives may affect the thermal stability of the material; and improper preparation conditions may lead to impedance mismatch, thereby impacting the final performance of the composites.

### 2.4. Hot Pressing

Hot pressing is a solid-state processing technique that densifies and shapes materials under the synergistic effect of high temperature and high pressure [27]. In the preparation of polyurethane composites, this method involves uniformly mixing prepolymers/polyols, isocyanate premixes (or partially reacted prepolymers), and fillers (such as fibers, nanoparticles, etc.), placing the mixture into a mold, and maintaining it under specific temperature and pressure conditions. This process promotes thorough cross-linking reactions, eliminates pores, and enhances interfacial bonding as well as mechanical properties [28].

**Figure 4:**
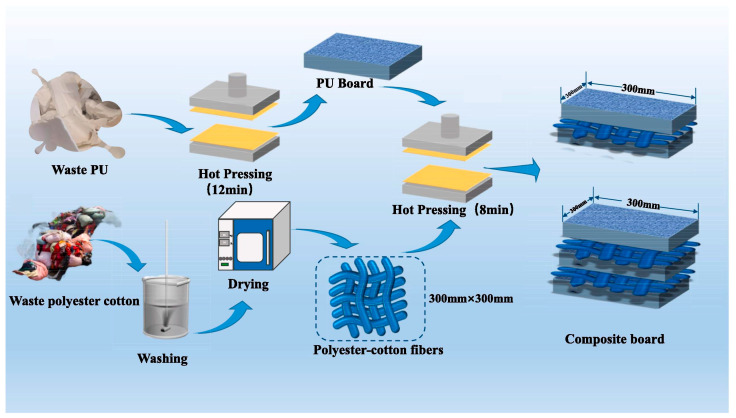
Schematic diagram of the fabrication process of WPC/WPU composites. Reprinted with permission from Ref. [29]. Copyright 2025 Elsevier.

Liang et al. [29] fabricated WPC/WPU composites by applying heat and pressure to tightly bond waste polyester-cotton fabric (WPC) and waste rigid polyurethane (WPU), as illustrated in Figure 4. The elevated temperature induced partial melting of WPC fibers and generated atomic or molecular induction effects at the interface with the WPU matrix, thereby enhancing interfacial adhesion. The applied pressure densified the material, reduced internal voids, and promoted the formation of an interdiffusion layer between WPC and WPU, alleviating stress concentration and further strengthening the overall interfacial bonding. This synergistic effect not only optimized the microstructure and crystallinity of the composite but also endowed the product with excellent mechanical properties, flame retardancy, thermal insulation, and sound absorption. Wang et al. [30] fabricated thin foam polyurethane composites with a “film-foam-film” (M-W-M) sandwich structure through filtration and hot-pressing techniques. The intermediate layer, composed of porous waste polyurethane foam, not only enhanced thermal insulation performance but also extended the propagation path of electromagnetic waves. Owing to this unique sandwich structure, the composites exhibited a high electromagnetic interference shielding effectiveness of up to 83.37 dB, along with excellent mechanical properties and remarkable infrared stealth capability. Wu et al. [31] employed a dynamic hot pressing (DHP) strategy to prepare polyurethane elastomers by injecting energy to promote molecular chain disentanglement and enhance hydrogen bonding interactions. This approach synergistically improved molecular chain mobility, energy dissipation capacity, and fracture resistance. The resulting products demonstrated significantly enhanced toughness, exceptional softness, ultrahigh stretchability, efficient energy dissipation, excellent self-healing properties, and good thermal stability.

**Table 1:** Preparation methods of polyurethane composites.

Preparation Method	Advantages	Disadvantages	References
In situ polymerization	Good interfacial bonding Simple synthesis Formation of 3D dynamic networks High electrical conductivity Easy recycling	Easy agglomeration of fillers Poor compatibility Decreased mechanical properties at high filler content	[14,15,16]
Solution blending	Homogeneous film preparation High stretchability Good toughness Strong self-healing Recyclability	Solvent residue Strict process control Temperature-limited self-healing Performance affected by improper concentration	[18,19,20]
Electrospinning	Controllable fiber morphology High porosity Large specific surface area Superhydrophilicity High breathability Antibacterial properties Good electromagnetic wave absorption	Certain components may reduce sensitivity Additives affect thermal stability Demanding preparation conditions	[24,25,26]
Hot pressing	Strong interfacial bonding Improved mechanical properties Flame retardancy, thermal insulation Electromagnetic shielding High thermal conductivity High-value utilization of waste	Sensitive to process parameters Possible sacrifice of thermal insulation Difficulty in controlling the uniformity of multilayer structures High temperature may cause structural changes	[29,30,31]

Hot pressing technology offers significant advantages in the synthesis of polyurethane composites. By precisely controlling temperature and pressure, it promotes strong interfacial bonding and the formation of interdiffusion layers between waste polyester-cotton fabric and the polyurethane matrix, thereby substantially enhancing mechanical properties, hydrophobicity, flame retardancy, thermal conductivity, flexibility, thermal stability, and electromagnetic interference performance of the composites. This enables high-value utilization of waste materials. However, this technique also has limitations. It is highly sensitive to process parameters; excessively high or low temperature and pressure may lead to performance degradation. The hot pressing process may partially compromise the inherent thermal insulation advantages of polyurethane or reduce its high-frequency sound absorption efficiency. Fabricating multilayer structures may present uniformity challenges, and elevated temperatures may induce chemical structural changes in polyurethane.

## 3. Classification of Polyurethane Composites

The properties of polyurethane composites depend not only on the synthesis methods but also on the type, content, and interfacial interactions of functional fillers. This section systematically analyzes the modification effects and action mechanisms of three mainstream types of functional fillers. Table 2 describes the comparison of advantages, disadvantages, and sensing mechanisms among three types of fillers: carbon-based materials, polymers, and metal nanoparticles/nanowires. Through Table 2, the differences between these three fillers can be clearly understood.

### 3.1. Carbon-Based Materials

By compounding polyurethane with carbon materials, its mechanical, thermal, electrical, and barrier properties can be significantly enhanced [32,33]. Carbon materials serving as fillers—such as graphene nanoplatelets (GNPs), carbon nanotubes (CNTs), and carbon fibers (CFs)—can modify the properties and applications of polyurethane composites [34]. The specific properties of these carbon-based filler-reinforced polyurethane composites are summarized in Table 3.

#### 3.1.1. Graphene (GNP)

Graphene is a two-dimensional carbon material characterized by a single atomic layer of carbon atoms arranged in a honeycomb lattice [35]. Its structural uniqueness stems from carbon atoms bonded via sp^2^ hybridization, forming a robust in-plane hexagonal framework accompanied by a delocalized π-electron system [36]. This configuration imparts exceptionally high in-plane stiffness, while the electronic behavior of graphene resembles that of massless Dirac fermions, resulting in ultrahigh electron mobility and excellent electrical conductivity [37]. Additionally, graphene exhibits outstanding thermal conductivity and distinctive optical absorption properties [38,39].

Chen et al. [40] combined graphene with ethyl cellulose and deposited graphene nanoplatelets onto a polyurethane sponge framework to form a continuous conductive network. The sensor exhibits sensitivities of 3.31 kPa^−1^ in region I (0–20 kPa), 0.26 kPa^−1^ in region II (20–100 kPa), and 0.02 kPa^−1^ in region III (100–400 kPa). The lateral compression sensitivities are 3.83 kPa^−1^ and 0.16 kPa^−1^, respectively, in the range of 0–25 kPa. Its response and recovery times are 80.6 ms and 124.0 ms (under a pressure of 40 Pa), respectively, with an ultra-wide detection range (from 1 kPa to 400 kPa). Cyclic stability reaches 2000 cycles under 20 kPa and 1100 cycles under 1 kPa, with negligible drift. The porous structure of the PU sponge, the uniform dispersion of Gr/EC nanosheets, and the strong interfacial adhesion to the sponge skeleton (facilitated by the bridging effect of EC) are key to achieving high performance. In particular, a graphene concentration of 30 mg/mL and a pore size of 20 ppi are identified as optimal to ensure a continuous conductive pathway and sufficient contact points, thereby optimizing the gauge factor and detection range. Zhang et al. [41] embedded two highly conductive materials—graphene and MXene—into a porous PU sponge skeleton and enhanced their stability with a PDMS adhesive. The sensor features high sensitivity (2.68 kPa^−1^ in the range of 0–15 kPa, 1.02 kPa^−1^ in 15–40 kPa, and 0.33 kPa^−1^ in 40–100 kPa), a wide detection range (0–100 kPa), fast response times (rise time of 61 ms and fall time of 84 ms; bending rise time of 40 ms and fall time of 72 ms; twisting rise time of 42 ms and fall time of 70 ms), low hysteresis (approximately 5.8% at 70% strain), and excellent cyclic stability (withstanding 8000 loading cycles with a response retention rate as high as approximately 92.8%). Cao et al. [42] modified and optimized a TPU matrix by introducing a binary plasticizer, while employing graphene oxide (GO) and MXene as conductive fillers. The abundant surface functional groups of GO formed hydrogen bonds with disodium terephthalate (DsT), effectively inhibiting the stacking of both GO and MXene and promoting interlayer electron transport. This sensor exhibits excellent piezoresistive performance, with a gauge factor (GF) of 5.56, a broad strain detection range of 0–240%, a response/recovery time of 21/19 ms, cyclic stability exceeding 8000 cycles, and a minimum detectable strain limit of 5‰. Meanwhile, it also displays a linear negative temperature coefficient (NTC) effect, with a TCR of –2.14 °C^−1^ in the range of 25–45 °C and –2.39 °C^−1^ in the range of 1–25 °C. The DsT-enhanced interfacial adhesion and hydrogen bonding network are crucial to its excellent cyclic stability, while the material’s energy dissipation characteristics (e.g., the gradually decreasing hysteresis after 20 stretching cycles, with residual strain within 25%) also affect the hysteresis performance.

Graphene can impart exceptional electrical conductivity to composites, enabling them to respond to external stimuli and demonstrating advantages such as high sensitivity, rapid response, mechanical strength, and structural stability in fields like flexible sensors. Graphene also offers tunable chemical and physical functionalities, ease of processing, and the ability to optimize material elasticity and recovery through the formation of three-dimensional porous structures. However, the application of graphene in composites also presents challenges. Graphene particles may detach from the matrix during long-term use, affecting the electrical conductivity and service life of the material. In certain cases, the incorporation of graphene may limit the detection range of composites or lead to uneven dispersion and agglomeration, thereby weakening interfacial bonding. Furthermore, insufficient interfacial adhesion and potential hysteresis effects in composites remain issues requiring further investigation and improvement.

#### 3.1.2. Carbon Nanotubes (CNTs)

Carbon nanotubes are hollow, tubular nanomaterials composed of carbon atoms arranged in sp^2^ hybridization [43]. Essentially rolled-up graphene sheets, they exhibit mechanical strength, electrical conductivity, and thermal properties far superior to those of traditional materials [44]. Based on the number of graphene layers, carbon nanotubes are primarily classified into two categories: single-walled carbon nanotubes (SWCNTs) and multi-walled carbon nanotubes (MWCNTs). SWCNTs consist of a single cylindrical graphene layer, typically with an outer diameter of 1–3 nm, and possess exceptional electrical and thermal conductivity, mechanical strength, flexibility, and ultra-lightweight characteristics [45]. MWCNTs are composed of multiple concentric graphene cylinders, resembling a Russian doll structure [46].

Chauhan et al. [47] utilized CNTs to participate in load bearing, bridging fibers, and matrix while enhancing interfacial interactions between the matrix and fillers. This significantly improved the puncture resistance, mechanical properties, and energy absorption capacity of the composites, successfully yielding polyurethane composites with excellent puncture resistance, outstanding energy absorption capability, reprocessability, and recyclability. Luo et al. [48] exploited the structural changes under strain of the conductive network formed by MWCNTs within a TPU matrix, which induced resistance variations enabling the composite to perceive deformation. The addition of 1-pyrenecarboxylic acid (PCA) significantly improves the dispersion of MWCNTs in the TPU matrix and enhances the interfacial interaction, thereby optimizing the conductive network. The composite exhibits an ultrahigh sensitivity (GFmax) of 10,279.95 at 300% tensile strain, with an elongation at break of 625.8 ± 12.3%. The sensor has fast response/recovery times (109 ms and 113 ms at 15% strain, respectively) and maintains good cyclic stability after 1200 cycles at 10% strain. Under 0–50% tensile-release strain, the mechanical hysteresis decreases significantly from 2.04 MJ/m^3^ after the first cycle to 0.65 MJ/m^3^ after the third cycle. Pan et al. [49] successfully prepared phosphorus-containing waterborne polyurethane/single-walled carbon nanotube composites via in situ polymerization, demonstrating excellent flame retardancy, outstanding electrical properties, remarkable EMI shielding durability, and good thermal stability. Through in situ polymerization, SWCNTs are uniformly dispersed in the WPU matrix, forming a three-dimensional conductive network. This material (with 15 wt% SWCNTs) exhibits excellent flame retardancy (damage length of 4.6 cm, limiting oxygen index of 34.1%) and EMI shielding effectiveness (21.8 dB at 10 GHz). Its electrical conductivity is 4.52 S cm^−1^. The EMI shielding durability is outstanding, with the conductivity decreasing by only 1.8% after 1000 bending cycles. The reflection values (4.6–19.3 dB) show a more pronounced increasing trend than the absorption values (4.9–9.6 dB), indicating that the enhanced conductivity plays a dominant role in the improvement of reflection.

When compounded with polyurethane, carbon nanotubes exhibit notable advantages alongside certain challenges. Their benefits primarily manifest in significantly enhancing the electrical conductivity, mechanical properties, and thermal stability of the composites. Carbon nanotubes possess excellent electrical conductivity, capable of imparting conductive capabilities to insulating polyurethane materials. The high strength and high modulus of carbon nanotubes can enhance the tensile strength and elongation at break of polyurethane, enabling superior mechanical performance while maintaining flexibility. Furthermore, carbon nanotubes can promote crystallization of polyurethane and synergize with flame retardants to improve the flame retardancy and thermal stability of the composites. However, carbon nanotubes also present certain drawbacks, primarily manifested as a tendency to agglomerate within the polymer matrix, compromising dispersion uniformity and conversely reducing the material’s energy absorption capacity. This limits the enhancement of composite properties, particularly at higher carbon nanotube contents, where agglomeration becomes more pronounced and may even lead to decreased mechanical performance.

#### 3.1.3. Carbon Fibers (CFs)

Carbon fiber is a fibrous material composed of carbon elements, widely used in the field of flexible sensors due to its properties such as high strength, high modulus, low density, high temperature resistance, and corrosion resistance [50,51]. CF consists of fiber bundles formed by carbon atom crystals with diameters of 5–10 μm and a carbon content exceeding 90% [52].

**Figure 5:**
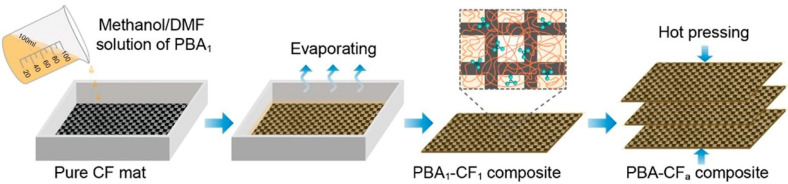
Schematic diagram of the fabrication process of PBA-CF composites. Reprinted with permission from Ref. [53]. Copyright 2025 Chinese Chemical Society.

Pu et al. [53] synthesized poly(phenylboronic acid)/carbon fiber composites (PBA-CF) with high strength, dynamic properties, and recyclability by incorporating poly(phenylboronic acid) networks with carbon fibers via hot pressing, as shown in Figure 5. The carbon fibers significantly enhanced the mechanical properties of the composite, providing a robust skeleton structure that tightened the connection between fiber bundles and substantially improved the fracture strength (757.3 MPa) of the composite. Liu et al. [54] prepared rGO/CFF/TPU porous composites by coating reduced graphene oxide (rGO) onto a carbon fiber felt (CFF) substrate and combining it with a TPU-DMF layer. The rGO acted synergistically to form an enhanced conductive network, while the hydrophobic surface and Marangoni stress contributed to the formation of a porous structure. The sensor has a response/recovery time of 60 ms, an ultrahigh sensitivity of 15.12 kPa^−1^ in the low-pressure range (<1 kPa), a wide detection range, and exhibits stable resistance hysteresis under 1000 loading cycles; long-term cycling (10,000 cycles) further demonstrates its mechanical-electrical durability. In addition, the composite achieves an EMI shielding effectiveness of 74 dB in the X-band, and an integrated neural network model enables material recognition accuracy exceeding 97.8%. Jian et al. [55] first prepared dynamically crosslinked polyurethane composites (V-PUs) via a solvent-free method using vanillin. They then used V-PU15 as the matrix resin and introduced carbon fibers to synthesize carbon fiber-reinforced polymer (CFRP) composites, successfully transferring the excellent properties of V-PUs to the CFRP. V-PU15, repaired at 120 °C for 1 h with the assistance of 5 L of DMF, achieves a repair efficiency of 91.95%. After four reprocessing cycles, the tensile strength and Young’s modulus remain at 87.53% and 88.02% of the original sample, respectively. The CFRP composite exhibits an interlaminar shear strength (ILSS) of 41 MPa, and the repaired ILSS reaches 85.34% of the original strength. Closed-loop recycling of carbon fibers and the resin matrix is achieved via solvent degradation. The composite prepared from the recycled materials shows a tensile strength of 298.32 MPa and a Young’s modulus of 22.28 GPa, corresponding to 92.23% and 88.28% of the original composite, respectively.

Carbon fiber is renowned for its lightweight and high-strength characteristics, endowing composites with exceptional mechanical properties, particularly in terms of strength and modulus. It also possesses good electrical conductivity and provides excellent electromagnetic interference (EMI) shielding effectiveness by forming efficient conductive networks that attenuate electromagnetic waves. Carbon fiber composites exhibit promising recyclability and reprocessability, with specific methods enabling closed-loop recovery of fibers and matrix resin while maintaining material performance. However, carbon fiber composites also have drawbacks. For instance, existing recycling methods are often costly and inefficient, making it difficult to achieve non-destructive recovery of components. Their interfacial stability may be insufficient, leading to reliability issues in sensing applications. The inherent rigidity of the fibers may cause discomfort or potential risks during use. Additionally, carbon fiber composites are sensitive to environmental conditions such as humidity, which may negatively impact their long-term performance. Finally, the preparation process for carbon fiber and its composites is often complex and may involve high-cost raw materials and environmentally unfriendly techniques, limiting their large-scale application.

### 3.2. Polymers

PU, owing to its excellent flexibility, biocompatibility, and processability, is frequently employed as a matrix material and combined with other polymers possessing specific sensing mechanisms to develop novel functional composites. Such composite materials typically leverage the mechanical properties of polyurethane alongside the functionalities of the other polymer to achieve diverse capabilities, including strain sensing. The specific properties of these polymer filler-reinforced polyurethane composites are summarized in Table 4.

#### 3.2.1. Rubber

Rubber is a polymer characterized by high elasticity, capable of undergoing reversible elastic deformation under substantial strain and rapidly recovering its original shape upon removal of external force [56]. This unique mechanical behavior renders it indispensable in fields such as flexible sensors [57]. Rubber is primarily classified into two major categories: natural rubber and synthetic rubber [58].

Ju et al. [59] synthesized rubber powder/polyurethane composites via in situ polymerization. After adding 20% rubber powder, the compressive strength of the composite (at 85% strain) increases to 588 kPa, and the fatigue life significantly improves from 10,258 cycles for pure PU to 45,987 cycles at a stress ratio of 0.6. The introduction of rubber powder, through its excellent energy absorption and dissipation characteristics, effectively dissipates mechanical energy under cyclic loading and makes the foam’s pore structure denser, reducing the pore size from 300–600 µm to 200–400 µm, thereby enhancing the toughness and fatigue resistance of the material and suppressing crack initiation and propagation. Suwan et al. [60] introduced silane groups into natural rubber-derived polyols, successfully preparing novel, hybrid, waterborne polyurethane composites with enhanced mechanical properties, improved thermal stability, increased hydrophobicity, and reduced water absorption. By chemically modifying natural rubber to produce bio-based polyols, the excellent elasticity and strength characteristics of natural rubber were successfully integrated into the final polyurethane material. Ahila et al. [61] incorporated hydroxylated crumb rubber particles as reactive fillers into polyurethane resin before polymerization. The strong interfacial adhesion resulting from polyurethane bond formation ultimately yielded a uniform and well-dispersed composite. The hydroxylated modified crumb rubber forms strong interfacial adhesion with the PU matrix, significantly increasing the tensile modulus of the composite by approximately 116% without compromising the tensile strength and elongation at break. When the content of hydroxylated crumb rubber increases to 20 wt%, the tensile modulus increases to 35 ± 4 MPa, but the tensile strength and elongation at break decrease to 1.62 ± 0.57 MPa and 37.9% ± 25.7%, respectively, indicating that a high filler loading may lead to particle agglomeration, thereby affecting the continuity of the matrix.

Rubber is a versatile material; through appropriate chemical modification, its drawbacks, such as poor compatibility with matrices, can be effectively overcome, thereby significantly enhancing the mechanical properties and functionality of composites without sacrificing other critical performance attributes. Rubber possesses excellent strength, elasticity, and outstanding energy absorption and dissipation characteristics, effectively improving the fatigue life and deformation resistance of materials, making it an ideal choice for various applications. As a renewable resource, the recycling and utilization of waste rubber also offers the advantage of cost-effectiveness. However, rubber also exhibits certain limitations. Unmodified rubber particles may present compatibility issues with certain polymer matrices, leading to poor interfacial adhesion. When the rubber content is excessively high, it may result in a non-uniform microstructure of the composite, reduced dispersion stability, and potentially decreased compressive and tensile strength, as well as material embrittlement.

#### 3.2.2. Polyvinyl Alcohol (PVA)

Polyvinyl alcohol (PVA) is a synthetic polymer produced through the polymerization of vinyl acetate followed by hydrolysis (or alcoholysis) reactions, with the chemical structural formula –(CH_2_–CHOH)n– [62]. PVA exhibits high hydrophilicity, capable of imparting excellent hydrophilic properties to composites. When combined with PU, these composite materials demonstrate significant application potential in fields such as flexible sensors and so on.

Zhao et al. [63] fabricated a highly hydrophilic composite nanofiber membrane by combining hydroxylated boron nitride nanosheets (OH-BNNSs) with waterborne polyurethane and PVA via electrospinning technology. The hydroxyl groups of PVA formed strong hydrogen bonds with waterborne polyurethane and OH-BNNSs; this interfacial interaction effectively reduced interfacial thermal resistance and significantly enhanced the thermal conductivity of the composite. The hydrogen bonding between PVA and OH-BNNSs also strengthened interfacial adhesion, enabling OH-BNNSs to effectively transmit stress. The membrane exhibits good mechanical properties, with yield strength, ultimate tensile strength, and elastic modulus reaching ≈3.85 MPa, ≈5.87 MPa, and ≈35.22 MPa, respectively. Putri et al. [64] employed polylactide (PLA)-derived polyurethane and MXene, incorporating PVA as a compatibilizer to stabilize the emulsion and promote hydrogen bonding interactions. The PLA-PU/MXene/PVA films with ratios of 6:4 and 7:3 achieve an optimal balance between tensile strength and flexibility. The 7:3 film exhibits a high gauge factor (GF) of 86.5, while the 6:4 film has a GF of 29.6, indicating that the 7:3 film possesses higher sensitivity but relatively lower cyclic stability (failing after 200 cycles, whereas the 6:4 film withstands 700 cycles). In terms of response time, the 6:4 film shows 479 ms and the 7:3 film shows 288 ms, with recovery times of 552 ms and 506 ms, respectively. Zheng et al. [65] synthesized a PVA-based hydrogel called MCPH and introduced it as a functional modifier into TPU, preparing high-performance TPU/MCPH composites. PVA provided the fundamental network of the hydrogel and formed dynamic boronic ester bonds with boric acid, significantly enhancing the energy dissipation, damping, water lubrication, and vibration and noise reduction performance of the TPU/MCPH composites.

PVA exhibits notable advantages and limitations when incorporated into PU composites. Its primary advantages include excellent hydrophilicity, biocompatibility, low cost, and ease of preparation. The hydroxyl groups on the PVA molecular chain enhance the material’s affinity for and storage capacity of water, promoting water lubrication effects, reducing the friction coefficient, improving hydrodynamic lubrication, and decreasing the frictional contact area. Furthermore, PVA fibers themselves possess high strength, high modulus, wear resistance, acid and alkali resistance, weather resistance, as well as non-toxic and environmentally friendly characteristics. These properties enable PVA to serve as physical crosslinking points, enhancing interfacial bonding through hydrogen bonding interactions and improving the mechanical strength, compressive properties, tensile properties, energy dissipation capacity, and self-recovery capability of the composite. However, when the PVA fiber content is too low, it may fail to effectively improve lubrication and may even exacerbate wear due to insufficient fibers to form an effective lubricating layer. Conversely, excessively high PVA content may lead to fiber aggregation, uneven dispersion, and the formation of stress concentrations, thereby compromising the mechanical properties of the composite and intensifying fatigue wear and material exfoliation.

#### 3.2.3. Cellulose

Cellulose is a naturally occurring polysaccharide biopolymer composed of D-glucose molecules as its fundamental building units [66]. These glucose molecules are linked through unique β(1→4)-glycosidic bonds, forming long, unbranched polymer chains [67]. This β(1→4) linkage configuration imparts a straight and rigid characteristic to cellulose chains, with cellulose also exhibiting high crystallinity, insolubility, resistance to enzymatic degradation, biocompatibility, and biodegradability [68].

**Figure 6:**
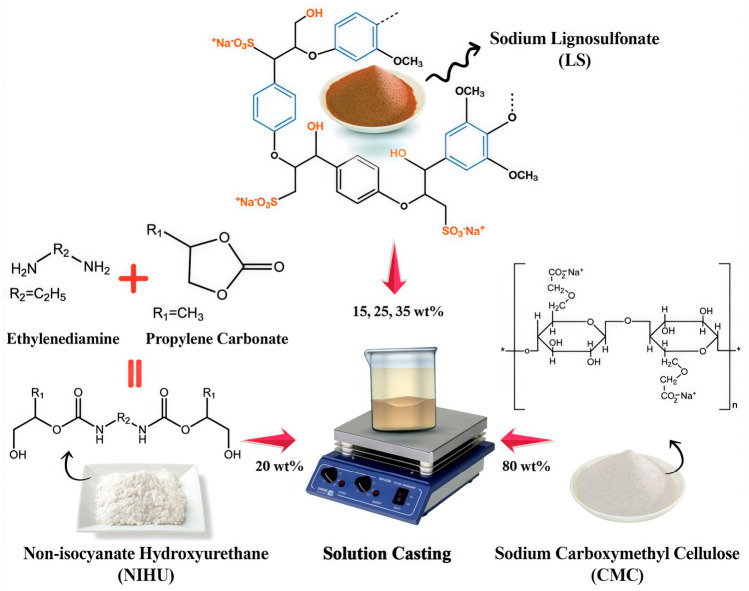
Schematic diagram of the fabrication process of LS, CMC, and NIHU composites. Reprinted with permission from Ref. [70]. Copyright 2026 Elsevier.

Zhang et al. [69] synthesized hydroxyethyl cellulose-polyurethane composites with excellent mechanical properties, efficient self-healing performance, and high moisture resistance through a “rigid-flexible synergy” strategy. Hydroxyethyl cellulose (HEC) was skillfully integrated into the flexible polyurethane matrix via chemical bonding, forming rigid HEC-rich domains that served as enhanced crosslinking points, significantly improving the mechanical strength of the material. The flexible polyurethane network was capable of driving these rigid HEC networks to participate in the self-healing process after damage (the self-healing efficiency is as high as 96.6%, which is 3.7 times that of the control group without disulfide bonds), overcoming the limitations imposed by the inherent rigidity of cellulose and achieving an efficient balance between material strength and self-healing capability. Oladzadabbasabadi et al. [70] introduced a composite film comprising sodium lignosulfonate (LS), non-isocyanate polyurethane (NIHU), and carboxymethyl cellulose (CMC), as illustrated in Figure 6. CMC provided the fundamental structural skeleton for the composite; its hydroxyl groups formed extensive hydrogen bonds with phenolic hydroxyl and sulfonic acid groups in NIHU and LS, promoting favorable interactions and dispersion among the components. The resulting composite film, while maintaining bio-based characteristics, exhibited enhanced mechanical properties, improved thermal stability, increased UV barrier capacity, enhanced antioxidant capability, and improved water vapor barrier performance. Melepalliyalil et al. [71] synthesized polyurethane composites by blending millable polyurethane (MPU) and cellulose acetate (CA) to form a polymer matrix, with quantitative incorporation of nano-hydroxyapatite. CA, as an integral component of the polymer matrix blended with MPU, imparted flexibility to the composite. The final product demonstrated excellent performance characteristics, including piezoelectric properties (the device generates a maximum output voltage of 3.4 V under finger tapping and a maximum power density of 16 μW/cm^2^ (at a load resistance of 1 MΩ)).

Cellulose, as a green and renewable bio-based material, exhibits significant advantages when compounded with polyurethane. These include its environmental friendliness, sustainability, and ability to enhance the mechanical strength, stiffness, and resistance to permanent deformation of composites by forming rigid domains that serve as multifunctional crosslinking points. Furthermore, cellulose derivatives such as cellulose acetate possess excellent biocompatibility, non-toxicity, environmental safety, and good film-forming properties, while also providing potential piezoelectric characteristics to composites. However, the inherent rigid structure of cellulose restricts molecular chain mobility, which may not only impede the self-healing properties of the material but also affect the overall flowability of polymer molecular chains and the exchange capacity of dynamic, reversible bonds. Additionally, cellulose-based materials typically exhibit high hygroscopicity, and their introduction may reduce the overall crystallinity of the composite.

### 3.3. Metal Nanoparticles/Nanowires

Metal nanoparticles (MNPs) are metallic crystals with dimensions in the nanometer range [72,73]. Owing to their unique size effects, surface effects, and quantum confinement effects, they exhibit physicochemical properties that are significantly distinct from those of bulk materials [74]. Metal nanowires are one-dimensional nanomaterials characterized by high aspect ratios, with diameters at the nanometer scale, while lengths can extend to micrometers or beyond [75]. These nanowires possess unique quantum confinement effects and an exceptionally high surface area-to-volume ratio, which can influence mechanical strength, optical properties, and electronic characteristics [76]. Silver nanoparticles (Ag NPs) and silver nanowires (AgNWs) are commonly employed as conductive fillers and have garnered considerable attention in polyurethane composites due to their high performance.

**Figure 7:**
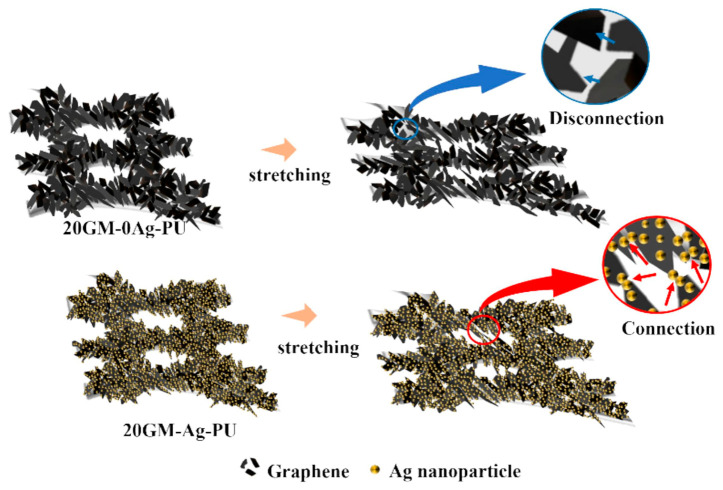
Comparison of the electrical conduction mechanisms of the GM-PU composite under tensile strain with and without Ag NPs. Reprinted with permission from Ref. [79]. Copyright 2025 Elsevier.

Materials such as Ag NPs and AgNWs can enhance the electrical conductivity of composites while also imparting antibacterial properties. Cao et al. [77] fabricated a 3D, wearable, piezoresistive sensor with waterproof and antibacterial functionalities by loading GO onto polyurethane foam, subsequently growing reduced graphene oxide-silver nanoparticles (rGO-Ag NPs) on the foam, and, finally, coating it with thermoplastic polyurethane. The resulting 3D TPU@rGO-Ag NPs@PU sensor demonstrates exceptional sensitivity (maximum gauge factor = 152.97 kPa^−1^), reliable dynamic stability (over 10,000 cycles), outstanding waterproof performance, high antibacterial activity (>99.9%), rapid response/recovery times (50 ms/40 ms), and a high sensitivity retention rate (approximately 90%). Wang et al. [78] developed a sensor comprising two layers of TPU nanofiber electrodes loaded with AgNWs and a sandpaper-like TPU/ionic liquid (IL) ionogel dielectric layer. The AgNWs form a highly interconnected three-dimensional (3D) network conductive structure within the TPU electrode, ensuring stable electrical conductivity under mechanical deformation and facilitating the formation and modulation of the electric double layer (EDL). Its capacitive sensing mechanism is based on the electric double layer (EDL) formed at the interface. The sandpaper-like rough structure and the easy deformability of the 3D mesh electrode significantly enhance the capacitance per unit area. The pressure sensing sensitivity reaches up to 106.01 kPa^−1^ (in the range of 0–4.3 Pa), with a resolution of 1.18 Pa, a response/recovery time of only 16 ms/25 ms, and excellent cyclic stability over 2000 cycles. Meanwhile, utilizing the mechanism of dielectric constant variation with temperature, the sensor exhibits a sensitivity of 28.1% °C^−1^ in the range of 20–40 °C, with a temperature resolution of 0.02 °C. Through signal processing, signal interference between pressure and temperature can be effectively eliminated, enabling accurate monitoring of pulse and thermal radiation. Huang et al. [79] synthesized a multifunctional, high-performance polyurethane composite (GM-PU) by exploiting the synergistic effect between graphene and silver nanoparticles within polyurethane foam. Silver nanoparticles were coated onto the graphene-modified polyurethane foam and uniformly distributed across the graphene surface, thereby further preventing agglomeration and realizing the synergistic interplay between graphene and silver nanoparticles, as illustrated in Figure 7. The piezoresistive strain sensing exhibits a GF of 66.3 in the strain range of 45–60%, a detection range of 0–60%, and good fatigue resistance over 250 cycles. The photothermal mechanism combines the high solar absorptivity of graphene (77.6%) with the plasmon resonance effect of Ag NPs, achieving a heating rate of 0.48 °C/s and a maximum temperature of 58.6 °C. In synergy with Ag NPs, it achieves 100% rapid bactericidal efficacy against *E. coli*.

Under applied pressure, Ag NPs effectively construct new charge transport pathways, which significantly enhance the sensitivity and conductive performance of sensors. Additionally, Ag NPs confer excellent antibacterial properties to sensors, a critical attribute for ensuring hygiene and health protection in the daily use of wearable sensing devices. Concurrently, Ag NPs effectively improve the electrical conductivity of reduced rGO and facilitate charge transport between graphene sheets.

## 4. Research Progress in Polyurethane-Based Sensors

Leveraging their excellent flexibility, biocompatibility, tunable molecular structures, and properties such as electrical conductivity, self-healing capability, and high sensitivity achieved through compositing with various functional materials, polyurethane composites demonstrate significant application potential in the field of flexible sensors. In view of this, this section will provide a detailed analysis of the specific application advancements of polyurethane composites in flexible sensors, focusing on the following four cutting-edge fields: environmentally friendly sensing, human motion monitoring, health monitoring, and bionic electronic skin.

### 4.1. Eco-Friendly, Flexible Sensors

Eco-friendly, flexible sensors represent a novel class of sensing devices that integrate environmental sustainability with mechanical flexibility, aiming to address the environmental pollution issues associated with traditional flexible electronic products [80]. These sensors are fabricated using biodegradable, renewable, or low-ecotoxicity materials in conjunction with environmentally benign manufacturing processes, enabling highly sensitive, low-hysteresis responses to various physical and chemical signals. Furthermore, they are capable of natural degradation or facile recycling upon the conclusion of their service life, thereby substantially mitigating the environmental impact of electronic waste [81].

**Figure 8:**
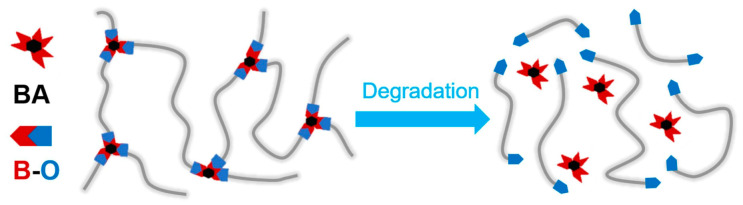
Schematic diagram of the degradation mechanism of IP-AD-BA1. Reprinted with permission from Ref. [82]. Copyright 2025 Elsevier.

Liang et al. [82] successfully synthesized a high-performance, readily degradable, and recyclable thermoset polyurethane composite (designated IP-AD-BA1). This sensor exhibits a dense porous architecture and the inherent elasticity of IP-AD-BA1, enabling rapid recovery to its original shape after compression. The sensor effectively detects both strain and stress in objects. IP-AD-BA1 demonstrates rapid recyclability under conditions of 110 °C and 15 MPa, while retaining its mechanical and thermal properties post-recycling. It can also be degraded in ethanol, facilitating the recovery of linear polyurethane oligomers, as illustrated in Figure 8. Through the percolation network formed by integrating carbon nanotubes (CNTs), this piezoresistive foam sensor can stably detect bending strains at various angles (30–90°) and pressures of up to 70 kPa. Experimental data show that after six recycling cycles, the material still maintains the end point of the hysteresis loop at around 100% under 500% cyclic strain, and its damping capacity remains stable, demonstrating the positive contribution of dynamic network rearrangement to cyclic stability. Ma et al. [83] synthesized a seawater-degradable polyurethane from bio-based monomers and successfully fabricated a high-performance flexible strain sensor through electrospinning. This sensor exhibits remarkable marine degradability, achieving a degradation rate of approximately 10% within 12 weeks in seawater, thereby effectively resolving the environmental pollution dilemma posed by conventional sensor materials that are resistant to degradation in natural environments. Functioning as a sensor, it also demonstrates outstanding electrical and mechanical performance, including an electrical conductivity of up to 19.08 S·m^−1^, stability and durability over 10,000 cycles of tensile testing, precise responsiveness to strains as low as 1%, and a rapid response time of 250 ms. Zhu et al. [84] converted waste polyethylene terephthalate (PET) into high-performance self-healing waterborne polyurethane, which was subsequently combined with polypyrrole to fabricate a flexible sensor. Its Tg is as low as −50 °C, endowing the material with good viscoelasticity and molecular chain mobility. By encapsulating a polypyrrole core at the gas–liquid interface to form a three-layer sandwich structure, the sensor enables monitoring of pressing and bending signals via a piezoresistive mechanism, with a response time of only 300 ms. Benefiting from the dynamic exchange of disulfide bonds and the interfacial adhesion of hydrogen bonds, the sensor achieves a tensile strength recovery rate of 93.72% after cutting and healing, while maintaining stable electrical performance (the U–I curve remains highly linear). No significant signal decay is observed over 50 repeated bending cycles, demonstrating the crucial role of the self-healing PU structure in extending the detection range and service life of flexible electronic devices. This strategy of valorizing waste PET not only offers a solution to plastic pollution but also lays a foundation for the development of long-lasting, low-cost, flexible electronic sensors, thereby presenting broad application prospects in fields such as environmental monitoring and protection.

Polyurethane composite-based sensors offer significant environmental advantages, manifested in their biodegradability, excellent mechanical properties, thermal stability, self-healing capacity, and recyclability, effectively reducing environmental pollution and conserving resources. They also feature high sensitivity, rapid response, and good cyclic stability, enabling precise monitoring of strain and stress. However, their limitations include potentially slow biodegradation rates under certain environmental conditions; complex synthesis processes that may escalate costs and difficulty; potential trade-offs where enhancing certain properties may compromise others; and the need for further in-depth investigation into their long-term stability and precision in complex environments.

### 4.2. Human Motion Monitoring Sensors

Human motion monitoring sensors are microelectronic devices or systems capable of real-time acquisition, recording, and analysis of physical parameters related to human motion [85,86]. Their core principle is grounded in physical sensing technology, which converts kinematic and dynamic information—such as the body’s spatial position, posture, acceleration, angular velocity, joint angles, gait cycle, and muscle activity—into processable, digital signals [87,88].

**Figure 9:**
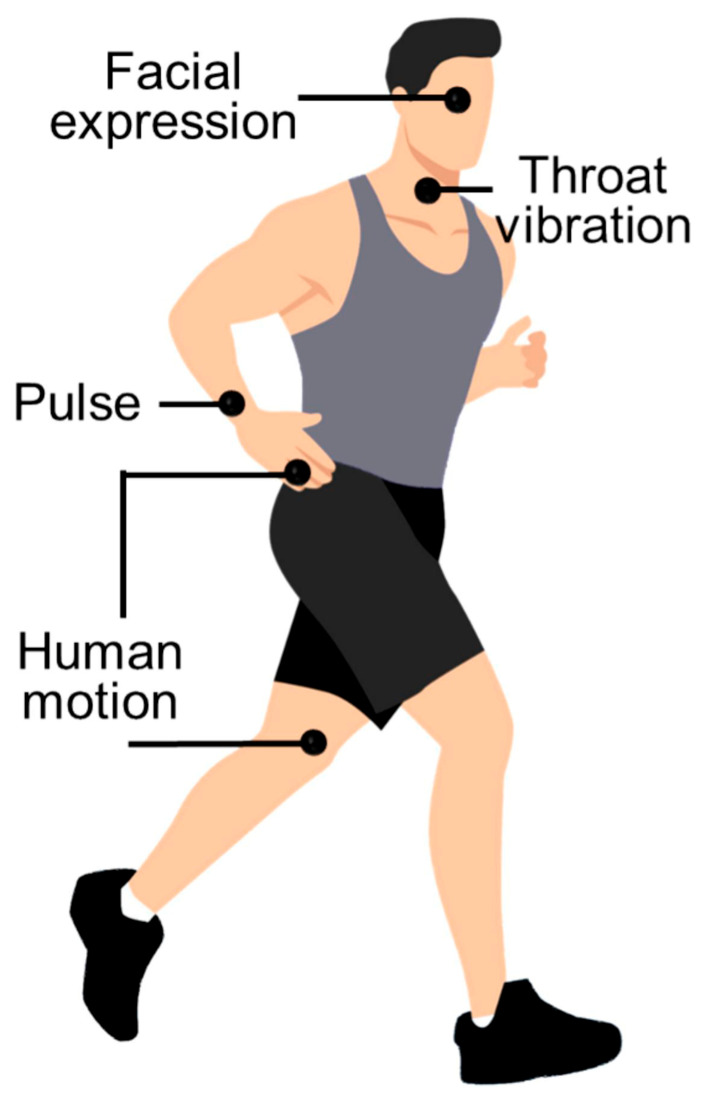
Schematic diagram of human motion detection. Reprinted with permission from Ref. [90]. Copyright 2025 Elsevier.

Wen et al. [89] developed a polyurethane composite with excellent mechanical properties through precise regulation of PEG molecular weight and HED content. As assessed by the gauge factor (GF), the sensor displayed high sensitivity, achieving a GF of 6.35 within the 0–15% strain range, 7.98 within the 25–60% strain range, and as high as 14.75 within the 70–90% strain range. The material exhibits a high tensile strength of 3.35 MPa and an elongation at break of 334% and can self-heal within 3 h at room temperature or in just 15 min at 60 °C. The sensor maintains excellent fatigue resistance after 1000 cyclic tensile tests. Moreover, with a relaxation period of 3 min, hydrogen bonds can re-form, effectively reducing the hysteresis effect in the stress-strain curve, thereby extending the service life and enhancing the reliability of the flexible sensor. Liu et al. [90] successfully synthesized a strain sensor based on a polyurethane membrane by constructing a dual-conductive layer composed of AgNPs and carbon nanotubes on the surface of an electrospun porous PU fiber membrane via a mussel-inspired adhesion mechanism. The sensor exhibited a low detection limit of 0.1%, a broad strain-sensing range from 0% to 687%, and remarkable durability over 6000 cycles. The loading-unloading curves of the sensor at 50% strain are almost overlapping, indicating very low hysteresis, with a response time of 190 ms and a recovery time of 200 ms. These characteristics enabled the successful application of the strain sensor in full-body human motion monitoring, including the detection of subtle physiological signals and large joint movements, as shown in Figure 9. Moreover, the high electrical conductivity and synergistic effect between AgNPs and CNTs conferred efficient photothermal conversion capability upon the sensor, facilitating its application in photothermal therapy at traditional Chinese medicine acupoints. The porous structure of the PU membrane endows it with good breathability; the water loss rate is comparable to that of pure PU fibrous membranes but six times higher than that of dense PU membranes. Combined with the antibacterial properties of AgNPs (antibacterial efficiency exceeding 99.9% against both Escherichia coli and Staphylococcus aureus), the wearing comfort is significantly enhanced. Li et al. [91] successfully fabricated a high-performance CNTs/SHPU fiber-based strain sensor by incorporating a self-healing polyurethane (SHPU) elastomer in combination with carbon nanotubes and a unique self-encapsulation structure. The material exhibits a tensile strength as high as 36.2 MPa, an elongation at break of 1086%, a stress self-healing efficiency of 94.4%, and a strain self-healing efficiency of 98.0%. The CNTs/SHPU fiber sensor has a tensile strength of 21.9 MPa and a strain of 1412%, and its electrical conductivity remains almost unchanged after cutting and self-healing. During 1000 cyclic tests, the relative resistance change gradually decreases and stabilizes within 0–500 s, which is mainly attributed to the redistribution of the CNT conductive network and the hysteresis effect caused by the viscoelasticity of the SHPU elastomer. Moreover, the self-encapsulating structure endows the CNTs/SHPU fiber with excellent solvent resistance and sensing stability, enabling its integration into wearable devices for human motion monitoring.

Polyurethane composite-based human motion monitoring sensors generally exhibit desirable flexibility, breathability, antibacterial properties, stretchability, and comfort, allowing them to accommodate a wide range of complex human movements and physiological signal monitoring requirements. Moreover, the incorporation of self-healing functionality can extend the service life of the sensors and enhance their reliability. Nevertheless, these sensors also present certain limitations. For example, self-healing efficiency may be constrained by environmental conditions, and achieving an optimal balance among high sensitivity, a broad sensing range, and good comfort remains challenging. Additionally, the inclusion of conductive materials may sometimes compromise the intrinsic mechanical properties of the substrate material, and during prolonged repeated use, signal stability may be affected by the reconstruction of the conductive network and the viscoelastic behavior of the material.

### 4.3. Health Monitoring Sensors

Health monitoring sensors are miniaturized, low-power, electronic sensing devices designed to collect human physiological parameters in real time, continuously, or intermittently and convert these biomedical signals into quantifiable and transmissible electrical signals or digital data for health monitoring and disease diagnosis [92,93]. Such sensors represent a key technology for enabling personalized medicine and remote health management [94].

**Figure 10:**
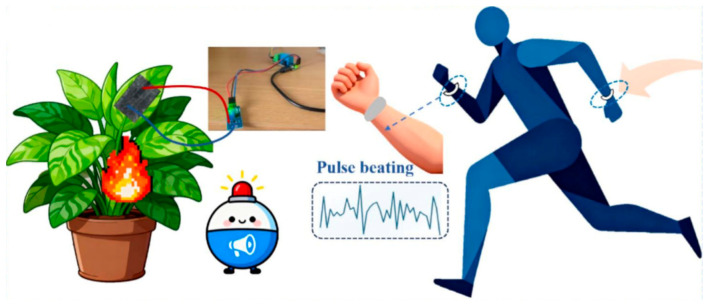
Schematic diagram of the sensor applied to health monitoring. Reprinted with permission from Ref. [95]. Copyright 2026 Elsevier.

Zhang et al. [95] successfully developed a flexible pressure-temperature dual-function sensing system capable of simultaneously achieving high-efficiency pressure and temperature signal detection, as shown in Figure 10. The as-fabricated sensor exhibited ultra-high sensitivity (GF ≈ 1200), a fast response time (110 ms), high durability, and stable performance without degradation even after up to 5000 motion cycles. This dual-function sensor enables precise temperature detection with a resolution of 1 °C. In practical applications, the sensor membrane exhibits significant breathability, with a water evaporation rate markedly faster than that of PDMS and PI membranes. It can be worn comfortably on the wrist and can monitor, in real time, subtle stress changes such as pulse, large stress changes such as finger bending, as well as temperature fluctuations such as body temperature. Wang et al. [96] developed a high-performance CNT/carbon black (CB)/TPU@PU flexible piezoresistive sensor for health monitoring applications. The sensor exhibits a sensitivity of 0.01 kPa^−1^ in both the low-pressure region (0–8 kPa) and the high-pressure region (8–23.3 kPa), along with a fast response time (119 ms), excellent washability, a rapid recovery time (59 ms), and outstanding repeatability (over 1000 cycles) and stability. TPU, serving as a binder, melts at 130 °C and firmly adheres CNT and CB to the sponge skeleton, endowing the sensor with remarkable washability. Under a pressure of 6.7 kPa, the ∆R/R_0_ of the CNT/CB/TPU@PU sensor decreases by 6% after one washing cycle and then stabilizes, whereas the CNT/CB@PU sensor without TPU shows an 18% decrease in ∆R/R_0_ after four washing cycles, confirming the enhancing effect of TPU on washability. Yu et al. [97] developed a self-healing sensor capable of real-time monitoring of temperature and pressure signals, which is highly suitable for human health monitoring. The sensor exhibited a gauge factor as high as 17.57 and a broad working range of 361.76%, with a tensile strength of up to 19.73 MPa. It can withstand 20,000 cycles under 50% strain. The sensor demonstrated negative temperature dependence in the range of 20–100 °C and a resolution of 0.01/°C between 36–40 °C. Solvent and heat treatments enabled a healing efficiency of up to 70.46% in terms of sensitivity.

Polyurethane composite-based health monitoring sensors offer advantages such as multifunctionality, high sensitivity, fast response, good durability and stability, self-healing capability, and good biocompatibility [98]. They can simultaneously monitor strain and temperature signals and adapt to various human activities and environmental changes [99,100]. However, their drawbacks include the potential aggregation of conductive fillers under certain composite formulations, which may lead to reduced sensitivity and a limited working range. Additionally, self-healing performance may degrade after the incorporation of nanomaterials, and baseline drift may occur during rapid stretching and releasing processes, potentially affecting the long-term accuracy of the signals.

### 4.4. Bionic Electronic Skin

In a broad sense, bionic electronic skin can be regarded as a type of flexible sensor system; however, its connotation extends far beyond that of a single flexible sensor [101]. It represents a complex, multifunctional integrated system designed to mimic, enhance, or even surpass the perceptual capabilities of biological skin [102].

**Figure 11:**
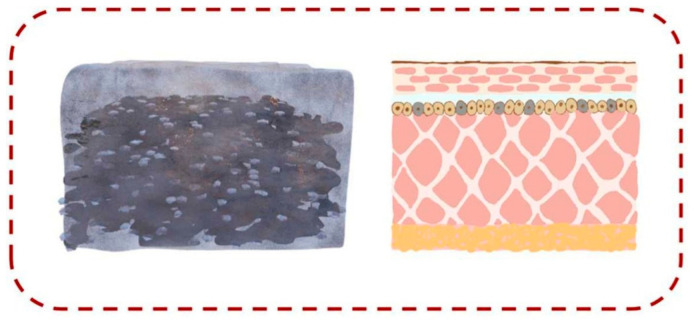
Schematic diagram of the sensor applied in bionic electronic skin. Reprinted with permission from Ref. [103]. Copyright 2026 Elsevier.

Zhou et al. [103] developed a liquid metal–graphene aerogel/multi-walled carbon nanotubes–polyurethane (LM-GA/MWCNTs–PU) composite flexible pressure sensor to simulate human skin, as shown in Figure 11. In this configuration, graphene aerogel incorporating liquid metal mimics subcutaneous tissue, MWCNTs enhance the conductivity and toughness of the matrix, and polyurethane improves the composite’s self-healing ability. As a piezoresistive sensor, it demonstrates high sensitivity (8.37 kPa^−1^), outstanding fatigue resistance (maintaining stability over at least 10,000 continuous cycles), and rapid response times (254/220 ms), enabling real-time tracking of human motion and signal transmission. Moreover, the sensor is low-cost, easy to fabricate, environmentally benign, and non-toxic. Zhang et al. [104] designed a self-healing, waterborne polyurethane composite with a rigid–soft phase-separated structure for bionic electronic skin. The composite exhibits excellent mechanical properties, with a tensile strength of 33.04 MPa, elongation at break of 954.79%, and toughness of 90.66 MJ m^−3^. When employed as an electronic epidermis, the composite conductor shows good self-healing and electrical response characteristics. The resulting flexible strain sensor can sensitively monitor human motion with stable and repeatable resistance responses. Shi et al. [105] synthesized a polyurethane composite containing disulfide bonds and multiple vinyl units as terminal groups for bionic electronic skin applications. After healing for 1 h and 1.5 h, the material achieved strengths of 104.22 ± 1.7% and 127.4 ± 12.6% of its initial value, respectively, and also exhibited thermally responsive shape memory functionality. The healable, stretchable sensor derived from this composite retained excellent sensing performance, effectively mimicking the sensory functions of natural skin. This work proposes a novel strategy for designing self-healing materials and holds substantial promise for advancing the development of future bionic skin.

Polyurethane composite-based bionic electronic skin offers significant advantages, including exceptional self-healing capability—enabled by dynamic covalent bonds and non-covalent interactions—which allows functional recovery after damage, thereby extending service life and improving reliability. Furthermore, such materials exhibit good flexibility, high sensitivity, fast response, and excellent cycling stability, making them highly promising for applications in electronic skin and tactile sensing. Nevertheless, certain drawbacks remain. Although the self-healing performance is superior, the mechanical strength and toughness of the material may be insufficient under high strain or after multiple healing cycles, potentially leading to performance degradation. In addition, the incorporation of conductive fillers (e.g., liquid metal or carbon nanotubes) may compromise self-healing efficiency, and issues related to the dispersion of liquid metal can result in unstable electrical conductivity within the composite.

## 5. Conclusions and Prospects

This paper provides a comprehensive review of fabrication strategies for polyurethane composites, including in situ polymerization [106], solution blending, electrospinning, and hot pressing. It systematically examines the effects of incorporating carbon-based materials (e.g., graphene, carbon nanotubes, carbon fibers), polymers (e.g., rubber, polyvinyl alcohol, cellulose), and metal nanoparticles/nanowires (e.g., silver nanoparticles and silver nanowires) into polyurethane matrices, highlighting both the resulting enhancements—such as electrical conductivity, mechanical properties, thermal stability, self-healing capability, and antibacterial activity—and the associated challenges. The review further summarizes recent progress in four major application domains of polyurethane-based sensors: eco-friendly sensing, human motion monitoring, health monitoring, and bionic electronic skin. Emphasis is placed on their outstanding performance in terms of high sensitivity, fast response, flexibility, biocompatibility, and self-healing functionality, while also addressing current limitations related to biodegradation rates, fabrication costs, trade-offs in material properties, and long-term operational stability.

Future research should focus on overcoming the existing challenges and exploring new application frontiers. Specifically, the development of novel, high-performance fillers is needed to resolve issues of uneven dispersion and weak interfacial adhesion, thereby enabling multifunctional, integrated sensor designs capable of meeting increasingly complex sensing demands. Further enhancement of self-healing efficiency and recyclability will contribute to improved sustainability and economic viability. For biomedical applications, a delicate balance between biocompatibility and biodegradability must be achieved. Additionally, the exploration of more efficient and low-cost intelligent manufacturing technologies is essential to facilitate large-scale production and commercialization. The deep integration of flexible sensors with cutting-edge technologies such as artificial intelligence and big data analytics holds great promise for enabling smarter data processing and application scenarios, potentially revolutionizing fields such as personalized health management and smart wearable devices. Sustained attention to the long-term stability and measurement accuracy of sensors will be critical to advancing the practical deployment of polyurethane composite-based flexible sensors.

Furthermore, there is an urgent need to establish standardized and normalized protocols for the performance evaluation of polyurethane-based flexible sensors. Currently, key metrics such as the gauge factor (GF) are frequently reported without considering essential parameters like Young‘s modulus, working strain range, hysteresis, response time, and cyclic stability. This fragmented approach hinders fair cross-comparison between highly deformable soft sensors (which prioritize compliance and large-strain capability) and stiffer, high-sensitivity systems (which often achieve high GF at the expense of stretchability). Developing unified evaluation frameworks that normalize these parameters—for example, by reporting sensitivity per unit strain energy or integrating hysteresis and recovery behavior into a figure of merit—will enable meaningful benchmarking and guide the rational design of sensors for specific applications.

## Figures and Tables

**Table 2:** Comparison of Advantages, Limitations, and Sensing Mechanisms of Main Fillers in Polyurethane Composites.

Filler Type	Advantages	Limitations	Sensing Mechanism	References
Carbon-Based Materials	Excellent electrical conductivity, high mechanical strength, good thermal stability, electromagnetic shielding properties, high sensitivity, fast response.	Prone to agglomeration, poor dispersion uniformity, insufficient interfacial bonding, potential performance degradation over long-term use, relatively high preparation cost.	Piezoresistive Effect: Structural changes in the conductive network under deformation lead to changes in electrical resistance.	[32,33,34,35,36,37,38,39,40,41,42,43,44,45,46,47,48,49,50,51,52,53,54,55]
Polymers	Good flexibility; biocompatibility; tunable mechanical properties; some possess self-healing, hydrophilic, or piezoelectric characteristics; renewable; cost-effective.	Compatibility issues with the matrix, high content can lead to non-uniform microstructure, may affect self-healing performance, high hygroscopicity, inherent rigidity restricts polymer chain mobility.	Piezoresistive Effect: Changes in the conductive filler network. Capacitive Effect: Polymer matrix acts as a dielectric layer, capacitance changes with deformation. Piezoelectric Effect: Certain polymers generate electrical charges under mechanical stress.	[56,57,58,59,60,61,62,63,64,65,66,67,68,69,70,71]
Metal Nanoparticles/Nanowires	Extremely high electrical conductivity, antibacterial properties, high sensitivity, wide measurement range, photothermal conversion capability.	Prone to agglomeration, poor dispersion stability, may reduce self-healing efficiency, mechanical properties may degrade after high strain or multiple healing cycles, rapid deformation can lead to signal drift.	Piezoresistive Effect: New charge transport pathways are constructed under applied pressure, and changes in the conductive network under deformation lead to changes in electrical resistance.	[72,73,74,75,76,77,78,79]

**Table 3:** The enhancement of the performance of polyurethane composites by carbon-based materials.

Carbon-Based Material	Performance Category	Specific Property	Enhancement Effect	References
Graphene (GNPs)	Sensitivity	Sensitivity of the flexible sensor	GF = 5.56	[42]
	Low detection limit	Minimum detected strain	0.5%	[42]
	Response time	Response/recovery time	80.6/124.0 ms (under a pressure of 40 Pa)	[40]
	Detection range	Ultra-wide detection range	0.001 MPa to 0.4 MPa	[40]
	Conductivity	Conductivity	Over 10,000 S·cm^−1^	[42]
	Response time	Response/recovery time	21/19 ms	[42]
	Biocompatibility	Biocompatibility	The mouse fibroblast cell line (L929) maintained a regular morphology after 24 h of incubation on the material	[42]
	Cyclic stability	Cyclic stability	8000 cycles	[41]
Carbon Nanotubes (CNTs)	Mechanical properties	Energy storage modulus	84.46%	[47]
		Tensile strength	67.15%	[47]
		Young modulus	65.7%	[47]
		Puncture resistance	38.66%	[47]
		Elongation at break	625.8 ± 12.3% (3 wt% MWCNT/PCA)	[48]
	Sensitivity	Strain sensor sensitivity	GF_max_ = 10,279.95 (0–300% strain)	[48]
	Conductivity	Electrical conductivity	4.52 S cm^−1^ (15 wt% SWCNTs)	[49]
	Electromagnetic shielding effectiveness	EMI shielding effectiveness	21.8 dB (15 wt% SWCNTs)	[49]
		EMI shielding durability	1.8% reduction (after 1000 bends)	[49]
Carbon Fibers (CFs)	Mechanical properties	Breaking strength	757.3 MPa	[53]
		Young modulus	35,700 MPa	[53]
	Recyclability	Closed-loop recycling capability	Tensile strength: 298.32 MPa (92.23% of the original composite material, after soaking in DMF at 100 °C for 3 h) Young’s modulus: 22.28 GPa (88.28% of the original composite material, after soaking in DMF at 100 °C for 3 h)	[55]
	Electromagnetic shielding effectiveness	EMI shielding effectiveness	Up to 74 dB (X-band)	[54]
	Self-healing performance	Repair efficiency	85.34% (interlaminar shear strength)	[55]

**Table 4:** The enhancement of polyurethane composite properties by polymer fillers.

Polymer Fillers	Performance Category	Specific Property	Enhancement Effect	References
Rubber	Mechanical Properties	Fatigue Life	From 10,258 cycles (pure PU) to 45,987 cycles (stress ratio 0.2), an increase of about 348%	[59]
		Compressive strength	From 5.6 ± 1.70 MPa (unfilled PU) to 6.55 ± 1.54 MPa, an increase of about 17%	[59]
		Tensile modulus	From 0.82 ± 0.13 MPa (unfilled PU) to 11.69 ± 3.38 MPa, an increase of about 1325%	[61]
		Abrasive resistance	From 20.76 mm^3^ (unfilled PU) to 18.62 mm^3^, a reduction of about 10.4%	[61]
	Surface performance	Hydrophobicity	When the content of MPTMS increased from 0.06 mol to 0.25 mol, the water contact Angle increased from 93° to 102°	[60]
Polyvinyl Alcohol (PVA)	Mechanical Properties	Yield strength	Reaches 3.85 MPa	[63]
		Ultimate tensile strength	Reaches 5.87 MPa	[63]
		Elasticity modulus	Reaches 35.22 MPa	[63]
		Flexibility	PLA-PU/MXene/PVA 1:1 (measured by elongation at break): 6.0%	[64]
		Mechanical strength	PLA-PU/MXene/PVA 6:4 (Measured by tensile strength): 5.84 MPa	[64]
		Compression modulus	From 50.55 MPa to 62.12 MPa, an increase of about 22.9%	[65]
		Compressive strength	From 20.14 MPa to 25.27 MPa, an increase of about 25.5%	[65]
		Tensile modulus	From 36.67 MPa to 53.12 MPa, an increase of about 44.8%	[65]
	Vibration-reducing performance	Vibration and noise reduction	The RMS value of the vibration signal: always remains between 0.103 m/s^2^ and 0.071 m/s^2^ (low and stable under all load conditions, Load dependence test, under water lubrication conditions; 5, 10, 15, 20, 30 N) The SPL of the noise signal: always maintained between 63.26 dB and 62.68 dB (low and stable under all load conditions, Load dependence test, under water lubrication conditions; 5, 10, 15, 20, 30 N)	[65]
	Electrical conductivity	Conductivity	Reaches 4.52 × 10^−5^ S/m	[64]
	Sensing property	Strain sensing sensitivity	The GF of the 7:3 film is 86.5 and the GF of the 6:4 film is 29.6	[64]
		Cyclic stability	The 6:4 film can withstand 700 bending cycles, while the 7:3 film fails after 200 cycles	[64]
Cellulose	Mechanical Properties	Tensile strength	MCH2: 0.925 MPa MCH4: 1.133 MPa MCH6: 1.188 MPa MCH8: 1.727 MPa MCH10: 1.241 MPa	[71]
		Young modulus	MCH2: 104 MPa MCH4: 156 MPa MCH6: 180 MPa MCH8: 443 MPa MCH10: 300 MPa	[71]
	Healing performance	Self-healing efficiency	Up to 96.6%	[69]
	Piezoelectric performance	Output Voltage from Finger Tapping	MCH2: 2.1 V MCH4: 2.7 V MCH6: 3.1 V MCH8:3.4 V (Maximum value) MCH10: 2.6 V	[71]
		Maximum Power Density	Under a load resistance of 1 MΩ, the maximum power density of 16 μW cm^−2^ was achieved	[71]
		Short-circuit Current	Under A load resistance of 1 MΩ, the maximum short-circuit current of 50 μA was achieved	[71]
	Barrier property	Water vapor permeability	Reduce by approximately 40%	[70]
		Ultraviolet ray blocking capacity	Almost completely blocked	[70]
	Antioxidant property	DPPH free radical scavenging activity	85%	[70]

## Data Availability

No new data was created or analyzed in this study. Data sharing is not applicable to this article.
